# Prognostic Value of Pericoronary Fat Attenuation Index on Computed Tomography for Hospitalization for Heart Failure

**DOI:** 10.1016/j.jacadv.2025.101685

**Published:** 2025-04-25

**Authors:** Mitsutaka Nakashima, Toru Miyoshi, Takahiro Nishihara, Takashi Miki, Kentaro Ejiri, Shohei Hara, Yoichi Takaya, Rie Nakayama, Keishi Ichikawa, Kazuhiro Osawa, Shinsuke Yuasa

**Affiliations:** a Department of Cardiovascular Medicine, Okayama University Graduate School of Medicine, Dentistry and Pharmaceutical Sciences, Okayama, Japan; b Department of General Internal Medicine 3, Kawasaki Medical School General Medicine Centre, Okayama, Japan

**Keywords:** coronary computed tomography angiography, fat attenuation index, heart failure, inflammation

## Abstract

**Background:**

Pericoronary fat attenuation index (FAI) assessed on computed tomography is associated with the inflammation of the pericoronary artery.

**Objectives:**

This study aimed to investigate whether pericoronary FAI predicts hospitalization for heart failure with preserved ejection fraction (HFpEF).

**Methods:**

This retrospective single-center study included 1,196 consecutive patients who underwent clinically indicated coronary computed tomography angiography (CCTA) and transthoracic echocardiography. We assessed the FAI of proximal 40-mm segments for each major epicardial coronary vessel. The primary outcome was the incidence of hospitalization for HFpEF. Patients were divided into groups based on the optimal cutoff value for predicting hospitalization for HFpEF by receiver operating characteristic curve analysis.

**Results:**

During a median follow-up of 4.3 years, 29 hospitalizations for HFpEF occurred. Multivariable Cox regression analysis revealed that a left anterior descending artery (LAD)-FAI ≥−63.4 HU and a left circumflex artery-FAI ≥−61.6 HU were significantly associated with hospitalization for HF after adjustment for age and sex (HR: 4.8; 95% CI: 2.1-10.8 and HR: 4.5; 95% CI: 2.1-9.4, respectively). The addition of LAD-FAI >−63.4 HU to a model incorporating other risk factors, including hypertension, estimated glomerular filtration rate <60 mL/min/1.73 m^2^, and significant stenosis on CCTA, increased the C-statistic for predicting hospitalization for HFpEF from 0.646 to 0.750 (*P* = 0.010).

**Conclusions:**

LAD- and left circumflex artery-FAI can predict hospitalization for HFpEF in patients undergoing clinically indicated CCTA. Pericoronary inflammation may be useful for identifying patients at high risk of developing HFpEF.

Heart failure with preserved ejection fraction (HFpEF) has become prevalent owing to the aging population.^1^ HFpEF has been reported to be associated with systemic inflammatory or metabolic disorders, which can directly impair the endothelial function of the coronary microvasculature.^2^, ^3^, ^4^, ^5^, ^6^ These disorders may alter the epicardial adipose tissue, further amplifying the effects of the systemic disorders on the underlying myocardium. The secretion of adipocytokines from dysfunctional epicardial adipose tissue leads to inflammation, microvascular dysfunction, and fibrosis of the underlying myocardium.^7^

Pericoronary fat attenuation index (FAI) assessed on coronary computed tomography angiography (CCTA), which reflects the perivascular fat inflammation of the coronary artery, is reported to be a risk factor for cardiac mortality or coronary artery disease (CAD).^8^, ^9^, ^10^, ^11^ In addition, increased pericoronary FAI is associated with coronary microvascular dysfunction in patients without obstructive CAD.^12^ The change in pericoronary FAI could be linked to myocardial dysfunction, leading to HFpEF.^3^^,^^13^ We reported that pericoronary FAI on computed tomography (CT) was significantly higher in patients with HFpEF than in those without HFpEF in a cross-sectional study.^14^ However, the relationship between pericoronary FAI and the development of HFpEF has not been elucidated.

In this study, we aimed to evaluate the prognostic value of pericoronary FAI in the incidence of hospitalization for HFpEF in patients undergoing clinically indicated CCTA.

## Methods

### Study population

This was a single-center retrospective study. Patients who underwent clinically indicated CCTA and transthoracic echocardiography (TTE) for suspected CAD from August 2011 to December 2016 at Okayama University Hospital were enrolled. Suspected CAD was defined by clinical symptoms such as chest pain and dyspnea or by abnormal electrocardiogram findings, including ST segment depression. Patients with histories of CAD, thoracic surgery, and hospitalization for HF, with left ventricular ejection fraction (LVEF) <50%, and undergoing hemodialysis were excluded. History of CAD was defined by a clinical history of known CAD, prior myocardial infarction, percutaneous coronary intervention, or coronary artery bypass grafting. We also excluded those with poor image quality for measurement of pericoronary FAI of any major epicardial coronary vessel and those lost to follow-up after CCTA.

This study conformed to the principles outlined in the Declaration of Helsinki and was approved by the ethics committee of Okayama University Graduate School of Medicine (approval number: 2203-024). The requirement for informed consent was waived because of the low-risk nature of the study and the inability to directly obtain consent from all enrolled patients. The study protocol was announced at the Okayama University Hospital, and patients were provided with the opportunity to withdraw from the study.

### Clinical outcomes

The primary outcome was the incidence of first hospitalization for HFpEF after CCTA. HF was diagnosed by experienced cardiovascular physicians according to clinical signs and symptoms. HFpEF was defined as HF with LVEF ≥50%. The secondary outcome was cardiovascular death (defined as death from CAD or other cardiovascular causes) after CCTA. Death from an unknown cause without obvious noncardiovascular cause was also included as a cardiovascular death in this study. The incidence of the outcomes was investigated through a retrospective review of medical records.

### CCTA, coronary artery calcium score, and pericardial fat volume

CT scans were performed using a 128-slice CT scanner (SOMATOM Definition Flash, Siemens Medical Solutions), as previously described.^15^ We evaluated the plaque characteristics in accordance with the Society of Cardiovascular Computed Tomography.^16^ High-risk plaque was defined as the presence of two or more features of positive remodeling, low-attenuation plaques, and spotty calcification. Positive remodeling was defined as a remodeling index >1.1, whereas low-attenuation plaques were defined as plaques with a CT attenuation number <30 HU. Spotty calcification was defined as a calcium burden length <1.5 times the vessel diameter and a width less than two-thirds the vessel diameter. Significant stenosis was defined as luminal narrowing >50% of any coronary artery. Coronary artery calcium in epicardial coronary arteries was assessed in 3.0-mm slices throughout the coronary artery regions, and the coronary artery calcium score was calculated using the Agatston method.^17^ Pericardial fat volume was quantified as the total volume of the tissues whose CT density ranged from −190 to −30 HU within the pericardium.^18^ These parameters were evaluated on a dedicated workstation (AZE Virtual Place; Canon Medical Systems Corporation).

### Pericoronary FAI

**Figure 1:**
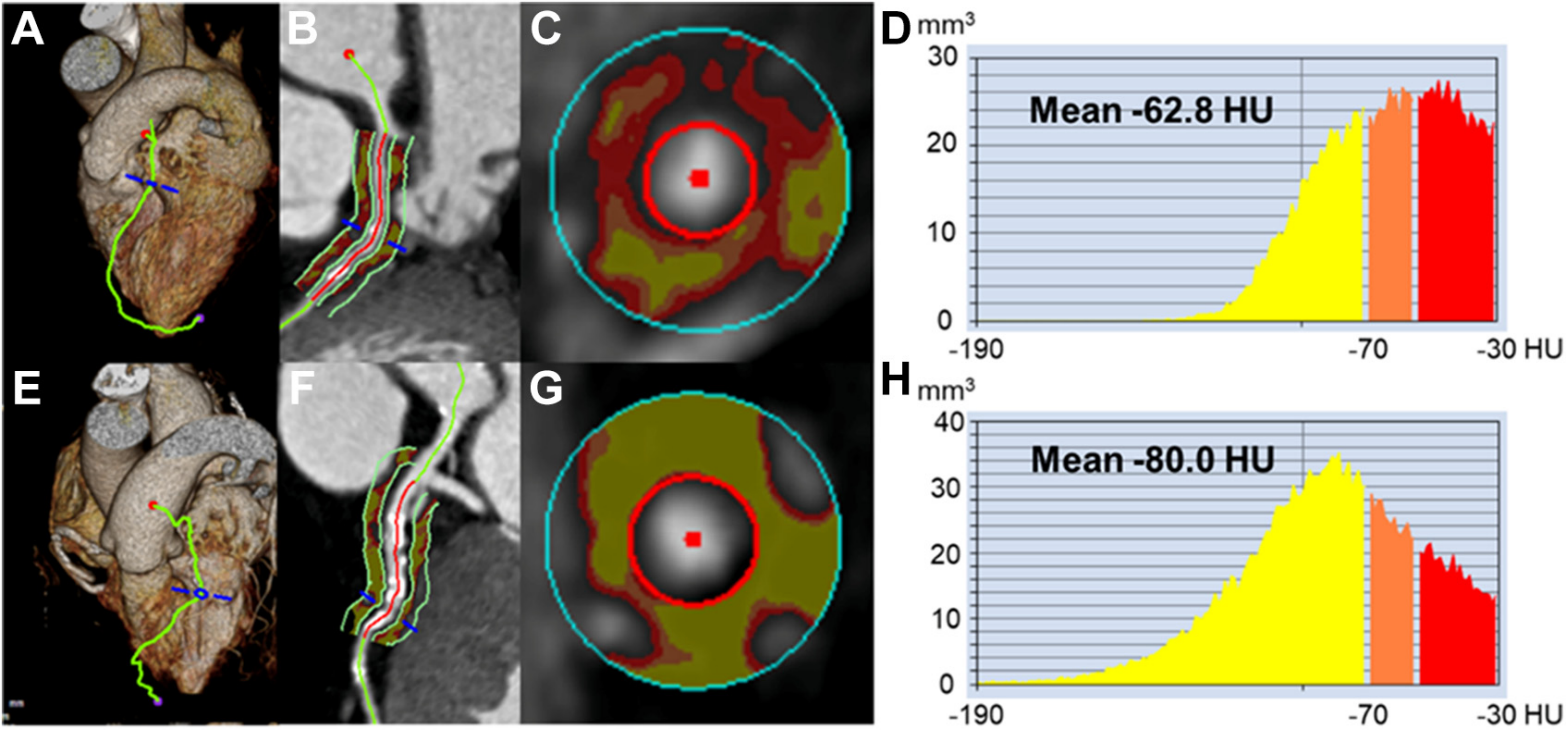
**Representative Case Showing Fat Attenuation Index by Coronary Computed Tomography Angiography** Three-dimensional reconstruction of the heart (A) and (E); pericoronary adipose tissue attenuation between −190 HU and −30 HU in the longitudinal view (B) and (F) and cross-sectional view (C) and (G); around the proximal 40 mm of the left anterior descending artery; histogram of CT attenuation within the traced area (D) and (H). A to D show pericoronary fat attenuation index in a case with the incidence of heart failure during follow-up. E to H show pericoronary fat attenuation index in a case without incidence of heart failure during follow-up. CT = computed tomography.

Pericoronary FAI was measured for all 3 major epicardial coronary vessels, right coronary artery (RCA), left anterior descending artery (LAD), and left circumflex artery (LCx), in all patients using a dedicated workstation (Aquarius iNuition Edition version 4.4.13, TeraRecon Inc). FAI was measured on the proximal 40-mm segments and traced with additional manual adjustments of the automatic delineation of each coronary vessel wall. The most proximal 10 mm of the RCA was excluded to avoid the effects of the aortic wall; thus, only the proximal 10 to 50 mm of the vessel was analyzed. Pericoronary fat was defined as the adipose tissue within a radial distance from the outer vessel wall equal to the diameter of the vessel.^9^ Adipose tissue was defined as all voxels with an attenuation between −190 HU and −30 HU. As shown in Figure 1, pericoronary FAI was automatically calculated as the mean CT attenuation value of pericoronary fat.^8^^,^^19^

### Transthoracic echocardiography

TTE using a 2.5- to 3.5-MHz probe with harmonic imaging (iE33 with an S5-1 probe, Philips Medical Systems, and Artida with a PST-25BT probe, Canon Medical Systems) was performed according to the American Society of Echocardiography guidelines.^20^ Two-dimensional measurements were performed and analyzed using standard views and techniques. The left atrial volume index (LAVI) was measured using B-mode presentation in the apical 2- and 4-chamber views. The left ventricular mass index (LVMI) was calculated as follows: LVMI = left ventricular mass/body surface area. The left ventricular mass was calculated using this formula: 0.8 × (1.04 × [(left ventricular end-diastolic diameter + intraventricular septum diameter + posterior wall diameter)^3^ – (left ventricular end-diastolic diameter)^3^]) + 0.6. LVMI was divided by the upper normal range: male, 115 g/m^2^; female, 95 g/m^2^.^20^ Body surface area was calculated using this formula: body weight^0.425^ × height^0.725^ × 0.007184.^21^ LVEF was measured by the modified Simpson technique using B-mode presentation in the apical 2- and 4-chamber views. We measured the peak early diastolic velocities (E) of left ventricular inflow and early diastolic myocardial velocities (e’). The ratio of E and e’ (E/e’) was calculated.

### Statistical analysis

Categorical variables are presented as numbers (%) and were compared using the chi-square test. Continuous variables with a normal distribution are presented as mean ± SD and were compared using Student’s *t*-test. Continuous variables without a normal distribution are presented as the median with 25th-75th percentiles (Q1-Q3) and were compared using the Mann–Whitney *U* test. Data normality was evaluated using the Shapiro–Wilk test. We assessed the association between pericoronary FAI and other clinical parameters, including TTE, using Spearman rank correlation (r) analyses.

For survival analysis, Harrell’s C-statistics were calculated as the area under the receiver operating characteristic (ROC) curves at the median follow-up period and presented with a 95% CI for the FAI of each major epicardial coronary vessel. The optimal cutoff value was defined as the point maximizing the Youden index. We estimated the cumulative incidence of HF hospitalization by HFpEF accounting noncardiovascular deaths as competing outcome with the comparison between the groups using Gray’s tests. Then, we quantified the association between pericoronary FAI and HF hospitalization and other outcomes using multivariable Cox regression models. To avoid overfitting, each model included limited potential confounding factors (≤3) for the risk of HFpEF: Model 1 as unadjusted model; Model 2 adjusted for age and sex; Model 3 adjusted for atrial fibrillation and estimated glomerular filtration rate (eGFR) as the presence of comorbidities that would affect hemodynamics; Model 4 adjusted for LVMI as the degree of left ventricular hypertrophy; Model 5 adjusted for LVEF and E/e’ as left ventricular systolic and diastolic function; Model 6 adjusted for the presence of significant stenosis and coronary artery calcium score measured by CCTA; and Model 7 adjusted for variables selected by a Least Absolute Shrinkage and Selection Operator (LASSO) regression model including variables in Models 2 to 6. In the Cox regression analysis, continuous variables were categorized using established cutoff or median values of this study population.^1^^,^^22^

We further assessed the incremental predictive value of pericoronary FAI over established predictors for HF hospitalization by HFpEF.^1^ The base model included variables selected by LASSO-Cox regression analysis from the following predictors: age (≥65 years), sex, higher body mass index (≥25 kg/m^2^), hypertension, diabetes mellitus, eGFR <60 mL/min/1.73 m^2^, and significant stenosis on CCTA. Incremental prognostic values were assessed using ROC curve analysis and global chi-square tests. C-statistics were compared using the Delong test, and the category-free net reclassification index was also calculated.

As sensitivity analysis, we also evaluated the association between pericoronary FAI and cardiovascular mortality. The same analytic method as the original analysis was applied. Statistical significance was set at *P* < 0.05. These analyses were performed using SPSS statistical software (version 25, IBM Corp.) and R, version 4.3.2 (R Foundation for Statistical Computing) software.

## Results

### Patient characteristics

**Figure 2:**
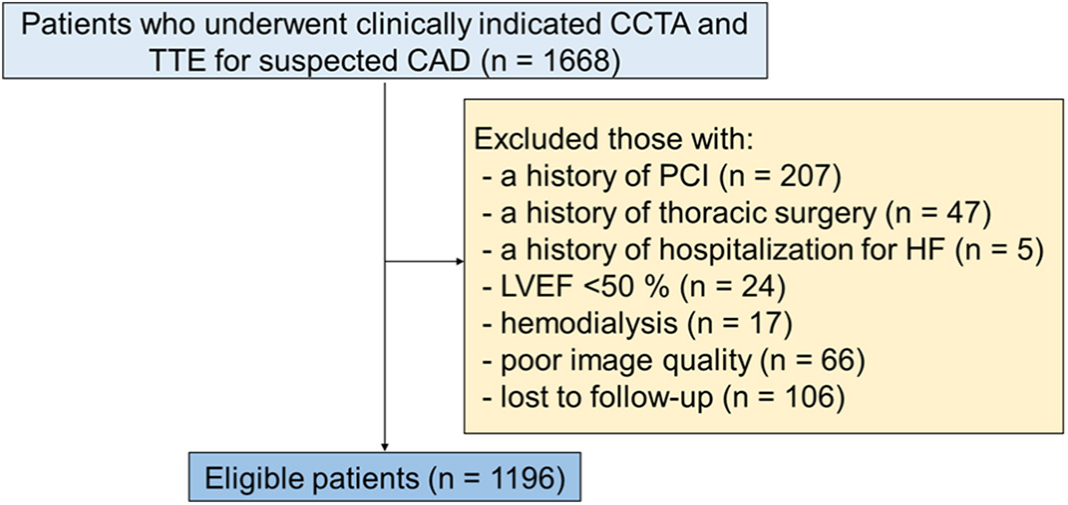
**Patient Flow Diagram** Among 1,670 patients without a history of CAD and who underwent CCTA and TTE for suspected CAD, those with histories of CAD, thoracic surgery, and hospitalization for HF, with LVEF <50%, undergoing emodialysis, with poor image quality for measurement of pericoronary FAI of any major epicardial coronary vessel, and lost to follow-up after the day of CCTA were excluded. A total of 1,196 patients were finally enrolled. CAD = coronary artery disease; CCTA = coronary computed tomography angiography; FAI = fat attenuation index; HF = heart failure; LVEF = left ventricular ejection fraction; PCI = percutaneous coronary intervention; TTE = transthoracic echocardiography.

Figure 2 presents the study flowchart. After excluding 472 patients who met the exclusion criteria, 1,196 patients (mean age: 63 ± 15 years; n = 700 males [58.6%]) were included in the analysis. Table 1 shows the clinical characteristics of the patients. The mean RCA-, LAD-, and LCx-FAI were −65.1 ± 8.3, −67.2 ± 7.3, and −64.6 ± 7.2, respectively. Significant correlations were observed between the FAI values of each major epicardial coronary vessel (Supplemental Figure 1).

**Table 1:** Baseline Clinical Characteristics of the Participants

	All Patients (N = 1,196)	LAD-FAI	
		≥−63.4 (n = 354)	<63.4 (n = 842)
Age, y	63 ± 15	64 ± 16	63 ± 15
Male	700 (58.6)	230 (65.0)	470 (55.9)
Body mass index, kg/m^2^	23.4 ± 3.9	22.1 ± 3.2	24.0 ± 4.1
Hypertension	678 (58.4)	197 (57.8)	481 (58.7)
Diabetes mellitus	346 (30.0)	92 (27.5)	254 (31.1)
Dyslipidemia	489 (43.0)	95 (28.7)	394 (48.8)
Atrial fibrillation	80 (6.7)	40 (11.3)	40 (4.8)
Current smoker	263 (22.5)	81 (23.5)	182 (22.1)
Medications			
β-blockers	208 (18.5)	71 (21.1)	137 (17.4)
ACEIs/ARBs	407 (36.3)	127 (37.8)	280 (35.7)
Statins	301 (26.9)	57 (17.0)	244 (31.1)
Laboratory data			
eGFR, mL/min/1.73 m^2^	72 ± 18	72 ± 19	72 ± 18
Total cholesterol, mg/dL	190 ± 37	184 ± 40	192 ± 36
LDL-cholesterol, mg/dL	113 ± 32	108 ± 33	115 ± 32
HDL-cholesterol, mg/dL	59 ± 17	60 ± 18	59 ± 16
Triglyceride, mg/dL	110 (80, 157)	96 (71, 134)	117 (84, 166)
BNP, pg/mL	32 (14, 77)	49 (22, 116)	26 (12, 61)
Echocardiography findings			
LAVI, mL/m^2^	37.3 ± 13.1	41.6 ± 16.0	35.5 ± 11.2
LVMI, g/m^2^	85.0 ± 23.4	90.2 ± 26.9	82.8 ± 21.4
LVEF, %	65.3 ± 7.4	64.0 ± 8.5	65.8 ± 6.9
E/e’	11.4 ± 5.3	12.5 ± 7.5	12.0 ± 4.0
Coronary CTA findings			
Significant stenosis	311 (26.0)	91 (25.8)	220 (26.2)
High-risk plaque	176 (14.7)	43 (12.5)	133 (16.3)
CACS	15 (0, 236)	24 (0, 243)	11 (0, 225)
Pericardial fat volume, mm^3^	120.3 ± 51.7	97.2 ± 45.8	120.3 ± 54.2

Values are mean ± SD, n (%), or median (25th, 75th percentile).

ACEI = angiotensin-converting enzyme inhibitor; ARB = angiotensin II receptor blockers; BNP = B-type natriuretic peptide; CACS = coronary artery calcium score; CTA = computed tomography angiography; E/e’ = early diastolic filling velocity/early diastolic velocity of the mitral annulus; eGFR = estimated glomerular filtration rate; FAI = fat attenuation index; HDL = high-density lipoprotein; LAD = left anterior descending artery; LAVI = left atrial volume index; LDL = low-density lipoprotein; LVEF = left ventricular ejection fraction; LVMI = left ventricular mass index.

### Correlation between pericoronary FAI and clinical parameters

As shown in Table 2, significant correlations of LAD- and LCx-FAI with age, male sex, body mass index, dyslipidemia, statin use, total cholesterol, low-density lipoprotein cholesterol, triglyceride, brain natriuretic peptide, LAVI, LVMI, and pericardial fat volume (all *P* < 0.001) and E/e’ (*P* = 0.006) were observed. LAD-FAI, but not LCx-FAI, was significantly correlated with LVEF. In contrast, RCA-FAI showed a reverse correlation with age and did not exhibit significant correlations with echocardiographic parameters, except for LAVI (Supplemental Table 1).

**Table 2:** Spearman Rank Correlation Between Pericoronary FAI and Various Clinical Parameters

	LAD-FAI		LCx-FAI	
	r	*P* Value	r	*P* Value
Age	0.095	0.001	0.126	<0.001
Male	0.125	<0.001	0.173	<0.001
BMI	−0.264	<0.001	−0.264	<0.001
Hypertension	0.018	0.55	0.039	0.19
Diabetes mellitus	−0.034	0.24	−0.020	0.51
Dyslipidemia	−0.195	<0.001	−0.183	<0.001
Current smoker	−0.008	0.79	0.044	0.14
β-blockers	0.042	0.16	−0.032	0.29
ACEIs/ARBs	−0.004	0.88	−0.027	0.38
Statin	−0.146	<0.001	−0.116	<0.001
eGFR	−0.039	0.18	−0.046	0.12
Total cholesterol	−0.122	<0.001	−0.179	<0.001
LDL-cholesterol	−0.133	<0.001	−0.161	<0.001
HDL-cholesterol	0.053	0.096	−0.014	0.67
Triglyceride	−0.182	<0.001	−0.197	<0.001
BNP	0.265	<0.001	0.250	<0.001
LAVI	0.217	<0.001	0.193	<0.001
LVMI	0.138	<0.001	0.188	<0.001
LVEF	−0.069	0.017	−0.044	0.14
E/e’	0.078	0.007	0.081	0.006
Significant stenosis	−0.011	0.71	−0.017	0.56
High-risk plaque	−0.070	0.016	−0.047	0.12
CACS	0.034	0.23	0.017	0.56
Pericardial fat volume	−0.242	<0.001	−0.273	<0.001

BMI = body mass index; LCx = left circumflex artery; other abbreviations as in Table 1.

### Influence of significant stenosis and early revascularization on the association between pericoronary FAI and incidence of hospitalization for hfpef

**Figure 3:**
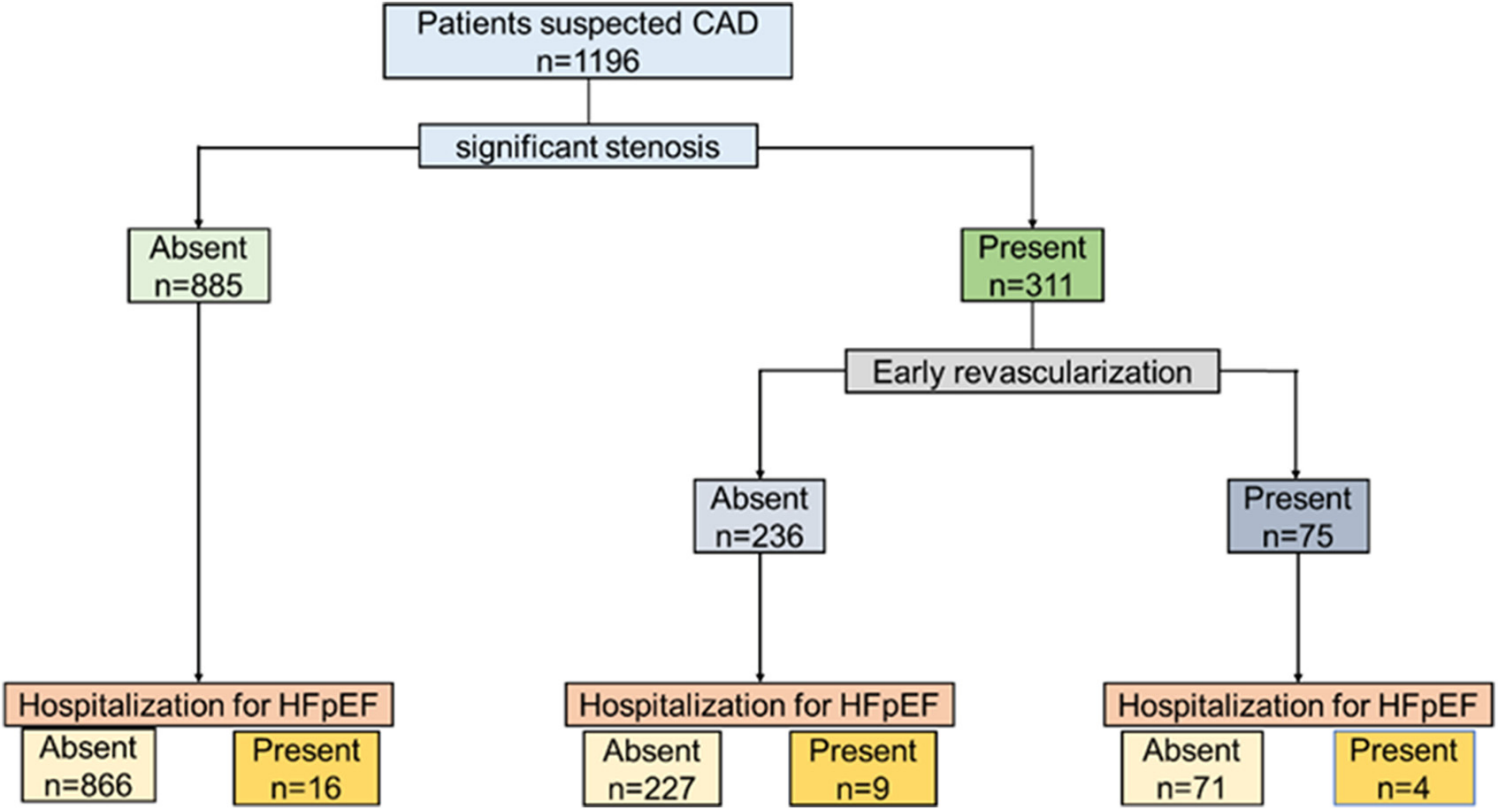
**Comparison of Pericoronary Fat Attenuation Index of the Left Anterior Descending Artery According to Significant Stenosis and Early Revascularization** The values of pericoronary FAI of the LAD were compared between patients with and without hospitalization for heart failure among those without significant stenosis. HFpEF = heart failure with preserved ejection fraction; LAD = left anterior descending artery; other abbreviations as in Figure 2.

Figure 3A shows a patient flow diagram according to the presence of significant stenosis and early revascularization for significant stenosis. Among 311 patients with significant stenosis, 75 patients underwent early revascularization (percutaneous coronary intervention, n = 72; coronary artery bypass grafting, n = 3).

### Association between pericoronary FAI AND hospitalization for HFpEF

**Figure 4:**
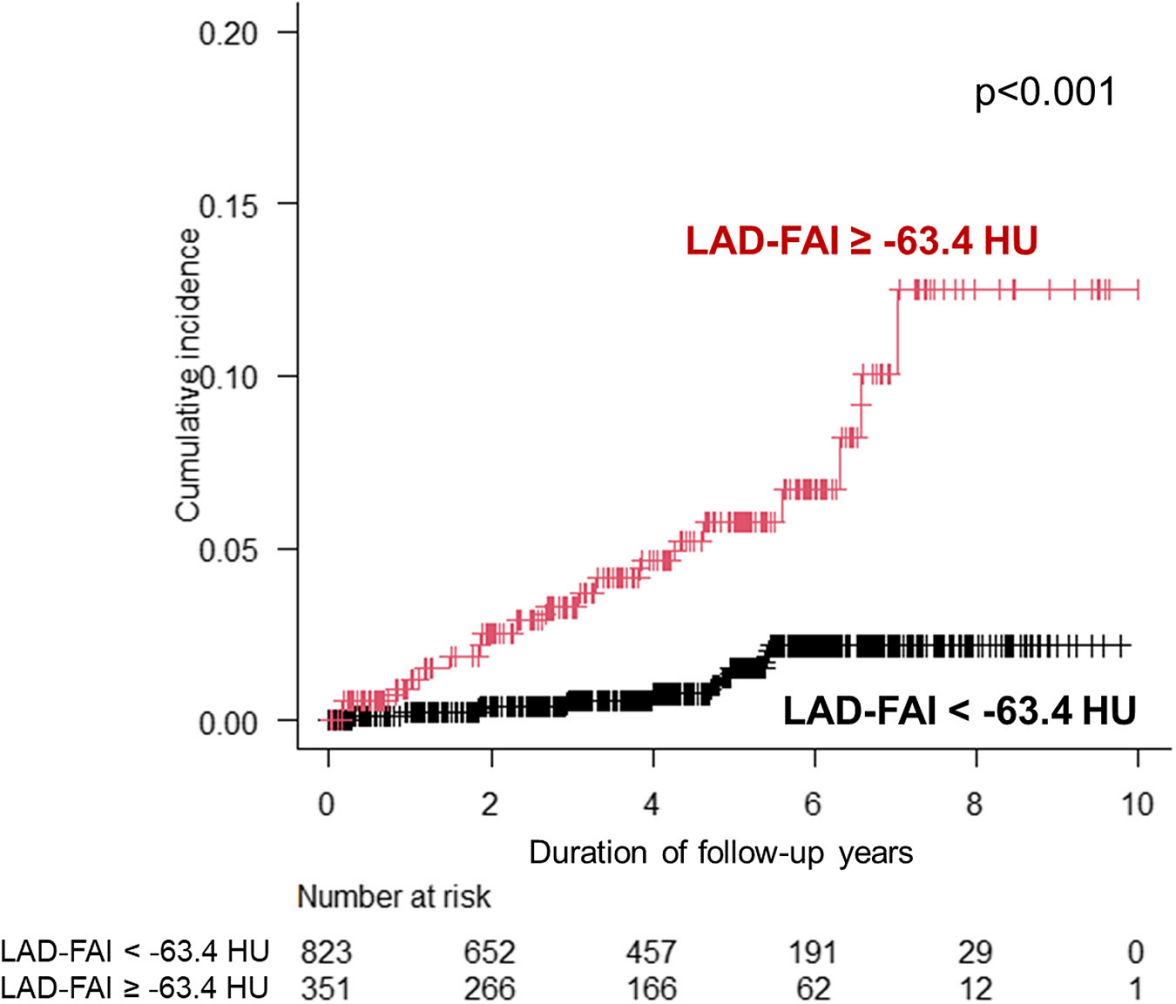
**The Cumulative Incidences of Hospitalization for Heart Failure With Preserved Ejection Fraction According to the Optimal Cutoff Value of Left Anterior Descending Artery-Fat Attenuation Index** Patients with a higher LAD-FAI had a significantly higher cumulative incidence of hospitalization for HFpEF than those with a lower LAD-FAI. Abbreviations as in Figures 1, 2, and 3.

During the follow-up period (median: 4.3 years, range: 2.4-5.8 years), hospitalization for HF occurred in 29 patients, all of whom had HFpEF. ROC curve analysis revealed that the C-statistics for predicting hospitalization for HFpEF were 0.769 (95% CI: 0.669-0.869) for LAD-FAI, 0.658 (95% CI: 0.512-0.805) for LCx-FAI, and 0.516 (95% CI: 0.378-0.655) for RCA-FAI. The optimal cutoff values for LAD-FAI and LCx-FAI were −63.4 HU and −61.6 HU, respectively. Figure 4 shows that the cumulative incidence of hospitalization for HFpEF was significantly higher in patients with high LAD-FAI than in those with low LAD-FAI. As shown in Table 3, multivariable Cox regression analyses demonstrated that LAD-FAI ≥−63.4 HU was significantly associated with the incidence of hospitalization for HFpEF after adjustment for age and sex (HR: 4.7; 95% CI: 2.2-10.2; *P* < 0.001), consistent with the results of other models, including Model 7, in which variables were selected using LASSO-Cox regression analysis: atrial fibrillation, eGFR, LVMI, LVEF, E/e’, significant stenosis on CCTA, and LAD-FAI.

**Table 3:** Cox Regression Analysis for Pericoronary FAI of the LAD and Hospitalization for HFpEF

	Hazard Ratio	95% CI	*P* Value	C-Index (95% CI)
LAD-FAI ≥−63.4 HU				
Model 1^a^	4.91	2.28-10.6	<0.001	0.696 (0.605-0.787)
Model 2^b^	4.71	2.18-10.2	<0.001	0.732 (0.631-0.833)
Model 3^c^	4.29	1.98-9.32	<0.001	0.766 (0.685-0.848)
Model 4^d^	4.43	2.04-9.65	<0.001	0.737 (0.648-0.826)
Model 5^e^	3.93	1.79-8.60	<0.001	0.747 (0.652-0.842)
Model 6^f^	4.79	2.22-10.30	<0.001	0.733 (0.618-0.849)
Model 7^g^	3.44	1.57-7.57	0.002	0.803 (0.716-0.890)

LVMI, LVEF, E/e’, and significant stenosis on CCTA.

CCTA = coronary computed tomography angiography; HFpEF = heart failure with preserved ejection fraction; LASSO = Least Absolute Shrinkage and Selection Operator; other abbreviations as in Table 1.

a Unadjusted.

b Adjusted for age and sex.

c Adjusted for atrial fibrillation and eGFR <60 mL/min/1.73 m^2^.

d Adjusted for LVMI.

e Adjusted for LVEF and E/e’.

f Adjusted for significant stenosis on CCTA and log-transformed CACS.

g Adjusted for variables selected by LASSO-Cox regression analysis: atrial fibrillation, eGFR <60 mL/min/1.73 m^2^.

### Prediction of future heart failure hospitalization by HFpEF

The addition of LAD-FAI ≥−63.4 HU to the base model including hypertension, eGFR <60 mL/min/1.73 m^2^, and significant stenosis on CCTA can improve the predictive performance of future HF hospitalization by HFpEF (the C-statistic improvement from 0.646 to 0.750 [*P* = 0.010]). Similarly, adding LAD-FAI ≥−63.4 HU to the base model significantly increased the global chi-square value and net reclassification index (8.0-14.6 and 0.368 [95% CI: 0.011-2.876]; *P* = 0.001 and *P* < 0.001, respectively). Similar results were obtained for LCx-FAI according to the optimal cutoff value (Supplemental Figure 2, and Supplemental Table 2). Multivariable Cox regression analyses demonstrated that LCx-FAI ≥−61.6 HU was significantly associated with the incidence of hospitalization for HFpEF after adjustment for age and sex (HR: 4.5; 95% CI: 2.1-9.4; *P* < 0.001), consistent with the results of other models.

### Sensitivity analysis

During the follow-up period, cardiovascular death occurred in 23 patients. Patients with LAD-FAI ≥−63.4 HU had a significantly higher cumulative incidence of cardiovascular death (*P* < 0.001); however, no significant association was observed with LCx-FAI (Figure 4, Supplemental Figure 3) (Central Illustration).

**Central Illustration:**
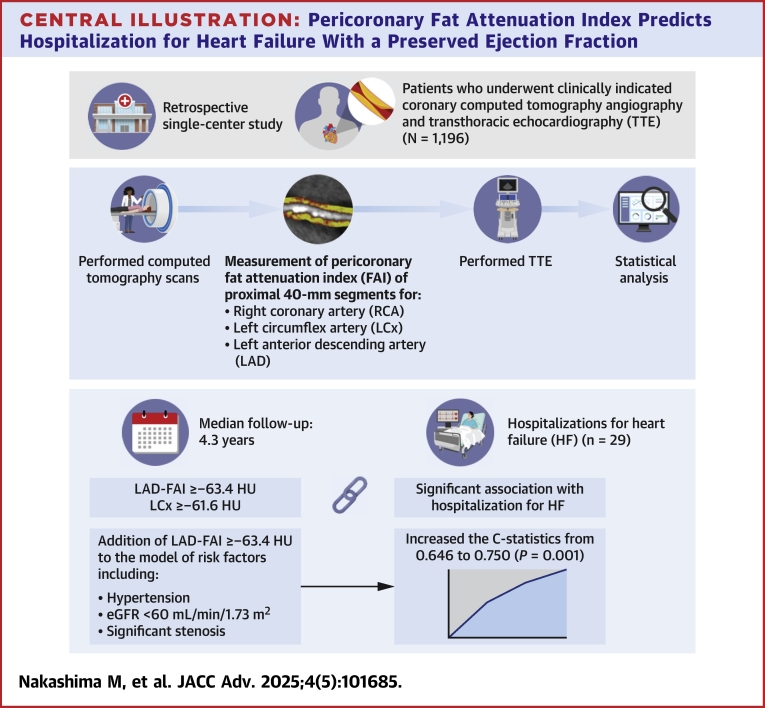
**Pericoronary Fat Attenuation Index Predicts Hospitalization for Heart Failure With a Preserved Ejection Fraction** eGFR = estimated glomerular filtration rate; other abbreviation as in Figure 1.

## Discussion

This study investigated the potential relationship between pericoronary FAI and hospitalization for HFpEF. High LAD- and LCx-FAI were associated with a higher incidence of hospitalization for HFpEF. Moreover, the addition of LAD-FAI to other risk factors for HF significantly improved the risk classification ability for future incidence of hospitalization for HFpEF in patients with suspected CAD. To the best of our knowledge, this is the first study to demonstrate that pericoronary FAI is a predictive marker of hospitalization for HFpEF.

Systemic and local inflammation play important roles in the development of HF, especially HFpEF.^2^^,^^4^, ^5^, ^6^ Patients with HFpEF have systemic complications or dysfunctions that cause chronic, low-grade inflammation, including aging, obesity, diabetes, hypertension, and chronic kidney disease.^23^ Pericoronary FAI represents early and chronic inflammation in pericoronary artery adipose tissue,^9^ indicating its role as a surrogate measure of coronary focal inflammation. Local cardiac inflammation leads to interstitial fibrosis or cardiac dysfunction.^3^^,^^5^ Meanwhile, a previous study reported a significant correlation with serum inflammatory mediators.^24^ Several serum biomarkers of systemic inflammation, such as interleukin-6 and soluble suppression of tumorigenesis-2, are reportedly associated with an increased risk of HF.^25^^,^^26^ These results suggest that high pericoronary FAI represents coronary focal and systemic inflammation, key contributors to HFpEF.

Pericoronary FAI is increased in case of epicardial CAD abnormalities, such as culprit lesions of acute coronary syndrome or obstructive CAD.^9^^,^^10^^,^^27^ Meanwhile, increased pericoronary FAI is reportedly associated with coronary microvascular dysfunction in patients without obstructive CAD.^28^ Coronary microvascular dysfunction leads to an increase in reactive oxygen species and a decrease in nitric oxide production, resulting in vascular endothelial dysfunction, cardiomyocyte hypertrophy, and stiffening.^3^ Recently, we presented a significant association between increased pericoronary FAI levels and peripheral endothelial dysfunction, as assessed by flow-mediated dilation of the brachial artery.^29^ Previous studies have revealed a significant correlation between flow-mediated dilation-assessed peripheral endothelial function and coronary artery endothelial function.^30^ Thus, high pericoronary FAI may reflect coronary microvascular dysfunction, a preceding factor of HFpEF. Additionally, patients who were hospitalized for HFpEF during the follow-up period exhibited significantly higher LAVI and E/e’ at baseline. These findings suggest that these patients may have underlying cardiac abnormalities, even in the absence of a history of HF hospitalization. We previously reported that patients with HFpEF had significantly higher FAI compared to those without HFpEF.^14^ Furthermore, this study revealed that LAD- and LCx-FAI were significantly correlated with LAVI and E/e’. Thus, high FAI may reflect potential cardiac abnormalities, such as left atrial dilation and left ventricular diastolic dysfunction, and may be associated with a worse prognosis in HF.

In a previous study, LAD- and RCA-FAI were associated with cardiac mortality.^8^ In another study, RCA-FAI, but not LAD- and LCx-FAI, was significantly associated with all-cause death and nonfatal myocardial infarction.^11^ Interestingly, this study illustrated that LAD- and LCx-FAI, but not RCA-FAI, were significantly associated with the incidence of hospitalization for HFpEF. In this study, LAD-FAI and LCx-FAI had a significant correlation with echocardiographic parameters associated with the incidence of hospitalization for HFpEF, whereas RCA-FAI correlated significantly only with LAVI. This suggests that LAD-FAI and LCx-FAI may be more important for the development of HFpEF than RCA-FAI. As LAD has the broadest myocardial perfusion lesion of the left ventricle among the 3 major epicardial coronary vessels, the incidence of HFpEF may be higher. Furthermore, in the multivariate Cox regression analysis including LVMI, LVEF, and E/e’, high pericoronary FAI was significantly associated with the incidence of hospitalization for HFpEF, indicating that pericoronary FAI relates to the development of HFpEF irrespective of baseline left ventricular function.

Anti-inflammatory therapy is a promising treatment for chronic HF. The canakinumab anti-inflammatory thrombosis outcomes (CANTOS) substudy examining the effect of canakinumab on HF showed improved maximal oxygen uptake and LVEF after 3 and 12 months, respectively, compared with placebo.^31^ Meanwhile, statins, eicosapentaenoic acid, and biological therapies, including antitumor necrosis factor α, have been shown to lower pericoronary FAI.^32^ Further studies are needed to investigate whether the effects of these therapies on pericoronary FAI could translate to a reduction in the hospitalization for HFpEF.

### Study limitations

This study has some limitations. First, this was a retrospective and single-center study that included Japanese patients only. It is unclear whether the results of this study may be applicable to other ethnic populations. Second, the number of outcomes was relatively small, thus limiting the use of several statistical analyses, such as stratification or adjustment of many variables for multivariable analysis. Third, we evaluated pericoronary FAI at a single time point; therefore, changes in pericoronary FAI after the day of CCTA remain unclear. Fourth, we did not obtain information about medication therapy after the day of CCTA despite reports that pericoronary FAI is influenced by the use of statins or biologic therapy for psoriasis.^32^^,^^33^ In addition, recently developed medications for the prevention or treatment of HFpEF, such as sodium-glucose cotransporter-2 inhibitors or angiotensin receptor-neprilysin inhibitors, may have influenced HFpEF progression during this study.^1^ Finally, this study comprised only Japanese patients for whom the prognostic value of pericoronary FAI is less reported. As Japanese people have a relatively small body size and a lower rate of obesity, the prognostic value of pericoronary FAI might differ from that of other populations, which warrants further investigation.

## Conclusions

LAD- and LCx-FAI predict hospitalization for HFpEF, and the addition of LAD-FAI to other risk factors for HF significantly improved the risk classification ability for future incidence of hospitalization for HFpEF in patients with suspected CAD. Pericoronary inflammation may be useful for identifying patients at high risk of HFpEF. However, the mechanism underlying pericoronary inflammation and HFpEF should be investigated in future research.

Perspectives **COMPETENCY IN MEDICAL KNOWLEDGE:** FAI for the LAD and LCx can predict hospitalization for HFpEF. The addition of LAD-FAI to other risk factors for HF significantly improved the risk classification ability for future incidence of hospitalization for HFpEF.

**TRANSLATIONAL OUTLOOK:** Pericoronary inflammation assessed on CT may be useful for identifying patients at high risk of HFpEF.

## Funding support and author disclosures

This study was supported by the 10.13039/501100001691Japan Society for the Promotion of Science KAKENHI (24K19032). The authors have reported that they have no relationships relevant to the contents of this paper to disclose.
