# Supplementary material for: Prognostic Value of Pericoronary Fat Attenuation Index on Computed Tomography for Hospitalization for Heart Failure
Source: JACC Adv. 2025 Apr 25;4(5):101685. doi: 10.1016/j.jacadv.2025.101685 (PMC12102526; doi:10.1016/j.jacadv.2025.101685)
Supplement: Supplemental_Material [file mmc1.docx]

**Supplemental Figure 1.** **Distribution and correlation of FAI across each major epicardial coronary vessel.**

(A) LAD-FAI and RCA-FAI, (B) LAD-FAI and LCx-FAI, (C) RCA-FAI and LCx-FAI.

FAI, fat attenuation index; LAD, left anterior descending artery; RCA, right coronary artery; LCx, left circumflex artery.

**
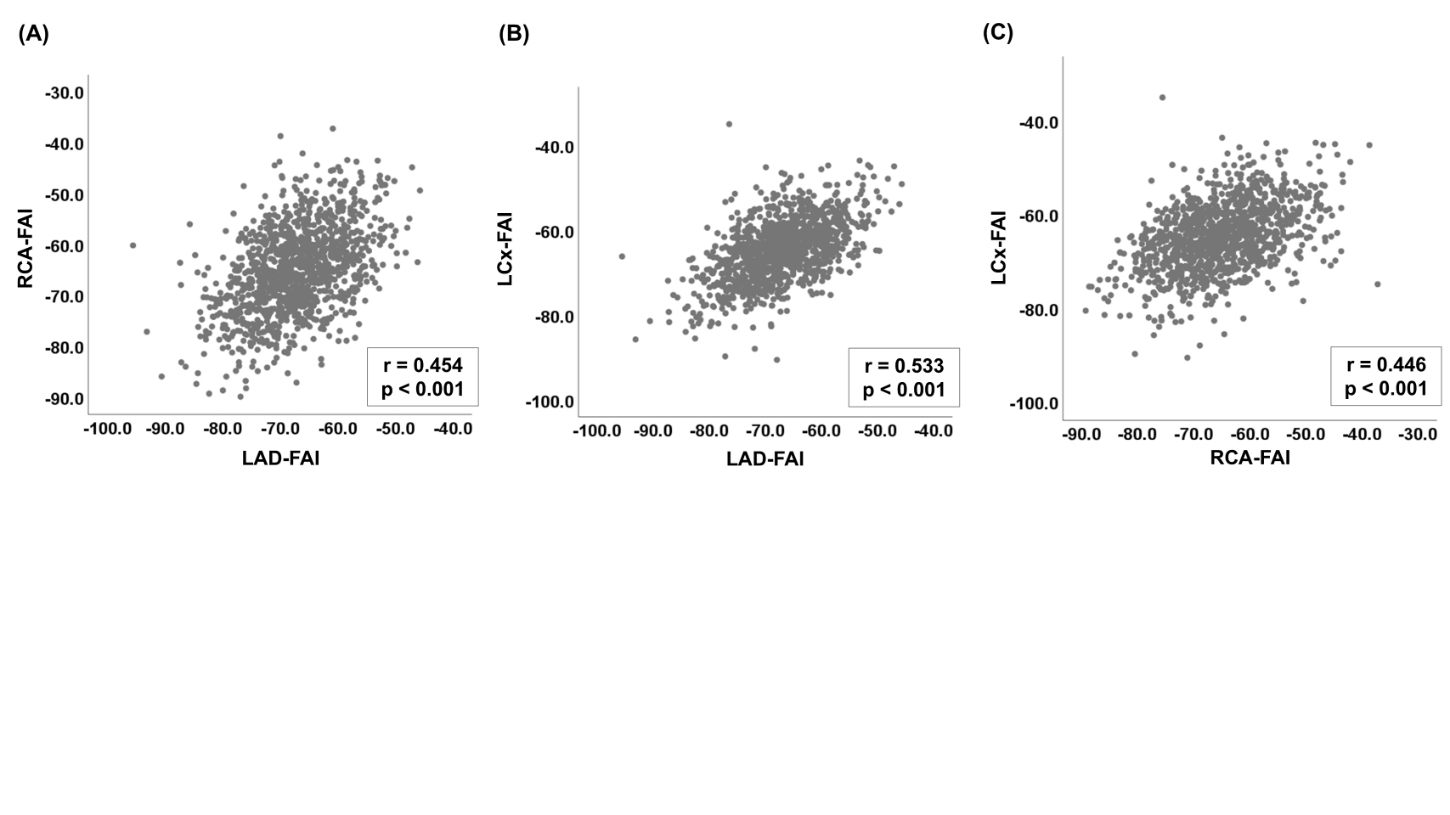
**

**Supplemental Table 1. Spearman rank correlation between RCA-FAI and various clinical parameters**

|  | RCA-FAI | |
| --- | --- | --- |
| Variable | *r* | *P* value |
| Age | -0.059 | 0.042 |
| Male | 0.007 | 0.81 |
| BMI | -0.249 | <0.001 |
| Hypertension | -0.107 | <0.001 |
| Diabetes mellitus | -0.090 | 0.002 |
| Dyslipidemia | -0.151 | <0.001 |
| Current Smoker | -0.005 | 0.86 |
| β-blockers | -0.055 | 0.069 |
| ACEIs/ARBs | -0.070 | 0.021 |
| Statin | -0.130 | <0.001 |
| eGFR | 0.034 | 0.25 |
| Total cholesterol | -0.084 | 0.006 |
| LDL-cholesterol | -0.103 | 0.001 |
| HDL-cholesterol | 0.103 | 0.001 |
| Triglyceride | -0.200 | <0.001 |
| BNP | 0.162 | <0.001 |
| LAVI | 0.093 | 0.002 |
| LVMI | -0.026 | 0.38 |
| LVEF | -0.034 | 0.24 |
| E/e’ | -0.042 | 0.15 |
| Significant stenosis | -0.094 | 0.001 |
| High-risk plaque | -0.049 | 0.096 |
| CACS | -0.096 | 0.001 |
| Pericardial fat volume | -0.389 | <0.001 |

FAI, fat attenuation index; LAD, left anterior descending artery; RCA, right coronary artery; BMI, body mass index; ACEI, angiotensin-converting enzyme inhibitor; ARB, angiotensin II receptor blockers; eGFR, estimated glomerular filtration rate; LDL, low-density lipoprotein; HDL, high-density lipoprotein; BNP, brain natriuretic peptide; LAVI, left atrial volume index; LVMI, left Ventricular mass index; LVEF, left ventricular ejection fraction; E/e’, early diastolic filling velocity/early diastolic velocity of the mitral annulus; CACS, coronary artery calcium score.

**Supplemental Table 2. Cox regression analysis for the perivascular FAI of LCx and hospitalization for HFpEF**

|  | Hazard ratio | 95% Confidence interval | *P* value | C-index (95% CI) |
| --- | --- | --- | --- | --- |
| LCx-FAI ≥-61.6 HU |  |  |  |  |
| Model 1^*^ | 4.09 | 1.90-8.79 | <0.001 | 0.677 (0.587-0.767) |
| Model 2^†^ | 3.94 | 1.82-8.51 | <0.001 | 0.710 (0.610-0.810) |
| Model 3^‡^ | 3.64 | 1.68-7.87 | 0.001 | 0.735 (0.631-0.839) |
| Model 4^§^ | 3.64 | 1.67-7.93 | 0.001 | 0.716 (0.628-0.804) |
| Model 5^\|\|^ | 3.42 | 1.57-7.46 | 0.002 | 0.733 (0.617-0.849) |
| Model 6^\|\|\|^ | 4.18 | 1.94-9.00 | <0.001 | 0.721 (0.613-0.829) |
| Model 7^\|\|\|\|^ | 3.22 | 1.46-7.10 | 0.004 | 0.778 (0.660-0.896) |

* Unadjusted

^†^ Adjusted for age and sex

^‡^ Adjusted for atrial fibrillation and eGFR

^§^ Adjusted for LVMI

^||^ Adjusted for LVEF and E/e’

^|||^ Adjusted for significant stenosis on CCTA and log-transformed CACS

^||||^ Adjusted for variables selected by LASSO-Cox regression analysis: atrial fibrillation, eGFR, LVMI, LVEF, E/e’, significant stenosis on CCTA

FAI, fat attenuation index; HU, Hounsfield units; LCx, left circumflex artery; eGFR, estimated glomerular filtration rate; LVMI, left Ventricular mass index; LVEF, left ventricular ejection fraction; E/e’, early diastolic filling velocity/early diastolic velocity of the mitral annulus; CACS, coronary artery calcium score; LASSO; Least absolute shrinkage and selection operator; BMI, body mass index; HFpEF, heart failure with preserved ejection fraction; CCTA, coronary computed tomography angiography.

**Supplemental Figure 2. The cumulative incidences of** **hospitalization for HFpEF according** **to the optimal cut-off value of LCx-FAI.**

LCx, left circumflex artery; FAI, fat attenuation index; HU, Hounsfield units.

**
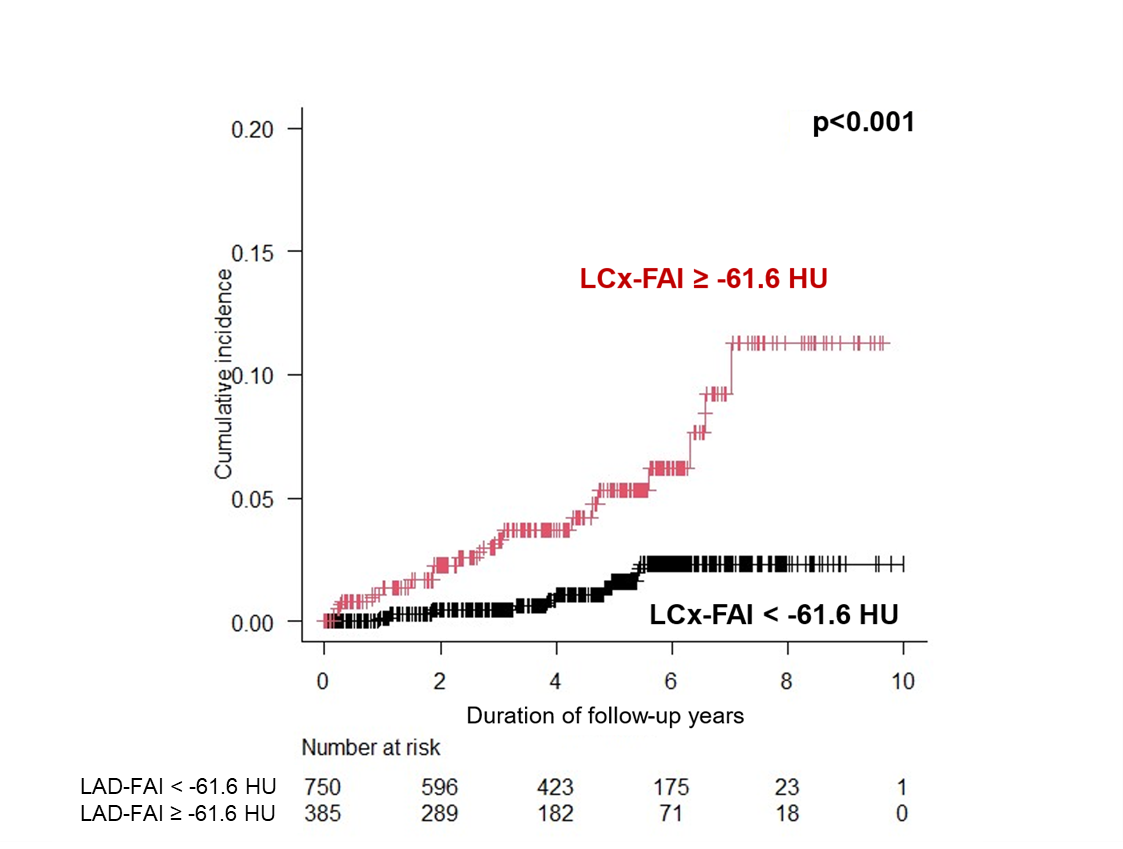
**

Patients with a higher LCx-FAI had a significantly higher incidence of hospitalization for HFpEF than those with a lower LCx-FAI.
